# A Potential Epigenetic Marker Mediating Serum Folate and Vitamin B_**12**_ Levels Contributes to the Risk of Ischemic Stroke

**DOI:** 10.1155/2015/167976

**Published:** 2015-02-01

**Authors:** Loo Keat Wei, Heidi Sutherland, Anthony Au, Emily Camilleri, Larisa M. Haupt, Siew Hua Gan, Lyn R. Griffiths

**Affiliations:** ^1^Centre for Biodiversity Research, Universiti Tunku Abdul Rahman, Bandar Barat, 31900 Kampar, Perak, Malaysia; ^2^Department of Biological Science, Faculty of Science, Universiti Tunku Abdul Rahman, Bandar Barat, 31900 Kampar, Perak, Malaysia; ^3^Genomics Research Centre, Institute of Health and Biomedical Innovation, Queensland University of Technology, Musk Avenue, Kelvin Grove, QLD 4059, Australia; ^4^Human Genome Centre, School of Medical Sciences, Universiti Sains Malaysia, 16150 Kubang Kerian, Kelantan, Malaysia

## Abstract

Stroke is a multifactorial disease that may be associated with aberrant DNA methylation profiles. We investigated epigenetic dysregulation for the methylenetetrahydrofolate reductase (*MTHFR*) gene among ischemic stroke patients. Cases and controls were recruited after obtaining signed written informed consents following a screening process against the inclusion/exclusion criteria. Serum vitamin profiles (folate, vitamin B_12_, and homocysteine) were determined using immunoassays. Methylation profiles for CpGs A and B in the *MTHFR* gene were determined using a bisulfite-pyrosequencing method. Methylation of *MTHFR* significantly increased the susceptibility risk for ischemic stroke. In particular, CpG A outperformed CpG B in mediating serum folate and vitamin B_12_ levels to increase ischemic stroke susceptibility risks by 4.73-fold. However, both CpGs A and B were not associated with serum homocysteine levels or ischemic stroke severity. CpG A is a potential epigenetic marker in mediating serum folate and vitamin B_12_ to contribute to ischemic stroke.

## 1. Introduction

Stroke is a multifactorial disease with 90% of cases being classified as ischemic stroke while hemorrhagic stroke makes up the remainder [1]. Emerging lines of evidence have suggested that aberrant DNA methylation may affect the vulnerability of central nervous system injury responses following ischemia [2, 3]. Dynamic interactions between DNA methylation and gene expression of pleiotropic factor thrombospondin 1 are associated with ischemic stroke [4]. DNA methylation of GNAS cluster [5] and sodium-potassium-chloride cotransporter types 1 and 2 [6] are associated with ischemic stroke.

Even though methylenetetrahydrofolate reductase (*MTHFR*) is actively involved in the generation of S-adenosylmethionine for methylation processes, to date, its methylation profiles have not been fully elucidated. Additionally, few data are available on the interaction between the differential DNA methylation profiles of* MTHFR* following cerebral ischemia. Castro et al. [7] demonstrated that hyperhomocysteinemia (levels higher than 75 *μ*mol/L) is associated with a 35% reduction in DNA methylation and increases the risk for atherosclerosis. This observation indicates that the increased risk of ischemic stroke may be explained, in part, by the effects of differential DNA methylation profiles on brain vulnerability in response to ischemic injury [4]. Hence, it is hypothesized that although the mechanisms underlying an increased risk of ischemic stroke have not been fully elucidated, differential* MTHFR* methylation profiles may better explain the pathogenesis of ischemic stroke. Therefore, in this study, we aim to investigate potential epigenetic dysregulation of* MTHFR* gene among ischemic stroke patients.

## 2. Materials and Methods

**Figure 1:**
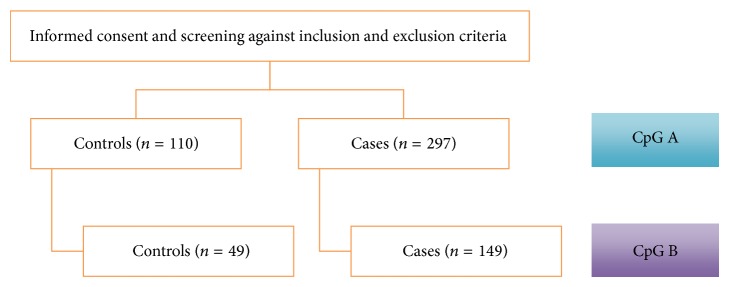
An overview of the study design.

This study was approved by the Institutional Ethics Review Committee of Universiti Sains Malaysia (reference number USMKK/PPP/JEPeM [231.1.(08)]) which complies with the Declaration of Helsinki. Signed written informed consent forms were obtained from each participant prior to their enrollments following screening against the inclusion and exclusion criteria. Cases were included if they were three generations of Malays, who were between 18 and 70 years old, who have shown occlusion, stenosis, and/or lesion on brain imaging system (computerized tomography scan or magnetic resonance imaging), and whose ischemic stroke severity has been assessed based on Modified Rankin Scale. Ischemic stroke patients were excluded if they participated in any on-going clinical trial study, they have taken any form of vitamins B supplementation six months prior to the study, they have brain tumors or other forms of cancer, or brain imaging has confirmed that patients suffer from intracerebral hemorrhage. Three generations of Malays with the same age group as cases with normal findings on medical history and physical examination were included as controls subjects. Healthy subjects who have positive family history of stroke, who have taken any form of vitamins B supplementation six months prior to the study, and who have medical illness that requires treatments were excluded as control subjects. It is important to note that positive association between CpG A methylation and ischemic stroke is further confirmed by 149 cases and 49 controls from the same population, and they are tested on CpG B (Figure 1). In addition, Modified Rankin Scale has been used as the ischemic stroke assessment tools because of the incomplete data of National Institute of Health Stroke Scale in all of the cases.

Fasting blood samples (6 mL) were collected in clot activator gel tube (Becton Dickinson) for the determination of serum vitamin profiles (serum homocysteine, folate, and vitamin B_12_ levels). Serum homocysteine levels were determined using fluorescence polarization immunoassay (Architect ci8200 Abbott, Illinois), while serum folate and vitamin B_12_ levels were determined using competitive immunoassay of direct chemiluminescence technology (ADVIA Centaur XP Immunoassay System, Siemens Healthcare). Venous blood samples (2 mL) were collected for DNA methylation profiling. DNA extraction, bisulphite treatment, and PCR-pyrosequencing were performed to determine methylation levels of CpGs A and B (Loo et al., unpublished data).

Statistical analysis was conducted using the R package version 3.0.3 (New Jersey, USA). The demographic characteristics of the subjects were analyzed using *t*-tests for continuous variables while chi-squared (*χ* ^2^) test was used for categorical variables. The distribution of serum vitamin profiles was investigated for normality using the Kolmogorov-Smirnov test. Pearson correlation coefficient (*r*) was used to establish the correlation between methylation and serum vitamin profiles followed by Bonferroni correction. Ischemic stroke severity was defined using a Modified Rankin Scale. Logistic regression models were used to estimate the odds ratios (ORs) and 95% confidence intervals (CI) for determining possible high risk methylation profiles for ischemic stroke and its severity. Additionally, potential confounders such as the age, genders, waist to stature, diabetes, hypertension, hypercholesterolemia, heart diseases, smoking, coffee drinking, tea drinking, and serum vitamin profiles were controlled in multivariate analysis.

## 3. Results

Cases and controls demonstrated similar waist to stature, age, and gender groupings (Table 1, all *P* > 0.05). However, the frequencies of diabetes, hypertension, hypercholesterolemia, heart diseases, smoking, coffee drinking, and tea drinking were significantly different when cases were compared with controls (Table 1, all *P* < 0.05). Correlation analysis demonstrated that the methylation levels in CpG A were positively correlated with serum folate (*r* = 0.106, *P* = 0.032) and vitamin B_12_ (*r* = 0.114, *P* = 0.022) levels but not with those in CpG B. Methylation profiles in both CpGs A and B were not significantly correlated with serum homocysteine levels. Following multivariate adjustment, CpG A methylation levels were shown to confer a significantly higher risk (by 4.73-fold) for ischemic stroke (95% CI: 2.56–8.75, *P* < 0.001) (Table 2). The susceptibility risk for CpG B levels was, however, not significantly associated with the risks for ischemic stroke (Table 2). In addition, CpG levels were not significantly associated with ischemic stroke severity following multivariate analysis (Table 3, *P* > 0.05).

## 4. Discussion

To our knowledge, this is the first study to investigate* MTHFR* methylation profiles among Asian ischemic stroke patients. Even though there is some support for a role of epigenetics in ischemic stroke risk [2, 7, 8], most studies [2, 8] have focused on increased susceptibility risk of ischemic stroke and long interspersed element-1 methylation. Similar to most epidemiological studies utilizing DNA from peripheral blood cells [8, 9], our study supports the notion that DNA is an informative determinant for epigenetic variation especially when brain cells are inaccessible [9]. We demonstrated that CpG A but not CpG B methylation levels were significantly associated with increased susceptibility risk for ischemic stroke.

*MTHFR* methylation profiles at CpG A were associated with serum folate and vitamin B_12_ levels and also with higher risks of ischemic stroke when compared to CpG B. Computational biology analyses have indicated that low density lysine 4 histone H3 trimethylation and incomplete RNA polymerase II in CpG B [10] may affect the transcriptional activity of* MTHFR*. Increased lysine 4 histone H3 methylation is an epigenetic mark for euchromatin, often accompanied by the presence of RNA polymerase II to act as an active promoter [11].

Several studies have demonstrated a link between vitamin profiles and DNA methylation indicating the pertinent role of one carbon metabolism in modulating DNA methylation [3, 9]. One carbon metabolism has been reported to be the main channel for methyl group donation at cellular levels [12] where a depletion of vitamin B_12_ or folate can reduce the bioavailability of S-adenosylmethionine [3]. Moreover, decreased bioavailability of S-adenosylmethionine can hamper the genome wide methylation process [2] and favor the reduction of lysine 4 of histone H3 methylation [3].

However, we have not established any correlation of* MTHFR *methylation with serum homocysteine levels suggesting that (1) an intergenic methylation of* MTHFR* may not play a major role in affecting the serum homocysteine levels and (2) serum homocysteine levels may not be an essential proxy for gene expression changes. Another possible explanation for the lack of correlation between* MTHFR* methylation and serum homocysteine levels could be partly due to the fact that serum S-adenosylhomocysteine levels may be a more sensitive marker as compared to serum homocysteine levels in determining the bioavailability of S-adenosylmethionine [13]. In fact, the nonsignificant correlation may also explain the nonsignificant correlation between* MTHFR* methylation and ischemic stroke severity. This is supported by the fact that homocysteine is actively involved in oxidative stress events which can affect vascular structure [14], thus worsening ischemic stroke severity [4, 15].

A recent report on the association between hemimethylated* MTHFR* gene and silenced* MTHFR* gene expression among end-stage renal disease patients [16] has further suggested a significant role of* MTHFR* promoter methylation on its gene expression levels. Therefore, future studies which investigate the effects of* MTHFR* methylation on gene expression changes are warranted for a better understanding of the epigenetic mechanisms of* MTHFR* in mediating serum vitamin profiles to contribute to ischemic stroke.

## 5. Conclusion

Methylation of* MTHFR* significantly increases susceptibility risk for ischemic stroke but does not affect ischemic stroke severity.* MTHFR* CpG A outperforms CpG B in mediating folate and vitamin B_12_ levels to increase ischemic stroke susceptibility by 4.73-fold; however, neither CpG was correlated with serum homocysteine levels.

## Figures and Tables

**Table 1:** Demographic characteristics of study subjects.

Parameters	CpG A		CpG B	
	Controls (*n* = 110)	Cases (*n* = 297)	Controls (*n* = 49)	Cases (*n* = 149)
Age (years)	52.74 ± 7.69	52.62 ± 8.83	51.12 ± 7.29	52.08 ± 8.21
Gender (male : female)	1.16	1.83	0.96	1.48
Waist to stature	0.52 ± 0.93	0.60 ± 1.36	0.51 ± 0.11	0.52 ± 0.11
Diabetes (%)	29.09	54.88^**^	24.49	65.10^**^
Hypertension (%)	9.09	76.77^**^	4.08	86.58^**^
Hypercholesterolemia (%)	9.09	71.38^**^	6.12	71.14^**^
Heart diseases (%)	11.82	23.91^*^	2.04	20.13^*^
Smoking (%)	29.09	43.43^**^	24.49	43.62^*^
Coffee drinking (%)	51.82	67.34^*^	44.90	64.43^*^
Tea drinking (%)	59.09	82.15^**^	63.27	85.91^*^

Values are the mean ± standard deviation from *t*-test for continuous variables and *χ* ^2^ test for categorical variables. ∗ and ∗∗ denote *P* < 0.05 and *P* < 0.001, respectively.

**Table 2:** Susceptible risk of DNA methylation on ischemic stroke.

CpGs	Crude			Adjusted		
	*β*	95% CI	*P* value	*β*	95% CI	*P* value^a^
A	3.85	2.72–5.45	<0.001	4.73	2.56–8.75	<0.001
B	1.38	1.08–1.77	0.011	0.90	0.56–1.45	0.975

^a^Multivariate analysis adjusted for age, genders, waist to stature, diabetes, hypertension, hypercholesterolemia, heart diseases, smoking, drinking coffee and/or tea, and serum vitamin profiles.

**Table 3:** Influence of DNA methylation on ischemic stroke severity.

CpGs	Crude			Adjusted		
	*β*	95% CI	*P* value	*β*	95% CI	*P* value^a^
A	−0.09	−0.15 to 0.13	0.601	−0.02	−0.16 to 0.12	0.743
B	0.02	−0.25 to 0.31	0.757	0.02	−0.71 to 0.85	0.946

^a^Multivariate analysis adjusted for age, genders, waist to stature, diabetes, hypertension, hypercholesterolemia, heart diseases, smoking, drinking coffee and/or tea, and serum vitamin profiles.
